# Modulation of p25 and inflammatory pathways by fisetin maintains cognitive function in Alzheimer’s disease transgenic mice

**DOI:** 10.1111/acel.12185

**Published:** 2013-12-17

**Authors:** Antonio Currais, Marguerite Prior, Richard Dargusch, Aaron Armando, Jennifer Ehren, David Schubert, Oswald Quehenberger, Pamela Maher

**Affiliations:** 1 The Salk Institute for Biological Studies10010 N. Torrey Pines Road, La Jolla, CA, 92037, USA; 2 Depatment of Medicine, University of California at San Diego9500 Gilman Drive #0601, La Jolla, CA, 92093-0601, USA

**Keywords:** astrogliosis, ERK, eicosanoid, lipoxygenase, oxidative stress, prostaglandin

## Abstract

Alzheimer’s disease (AD) is the most common type of dementia. It is the only one of the top ten causes of death in the USA for which prevention strategies have not been developed. Although AD has traditionally been associated with the deposition of amyloid β plaques and tau tangles, it is becoming increasingly clear that it involves disruptions in multiple cellular systems. Therefore, it is unlikely that hitting a single target will result in significant benefits to patients with AD. An alternative approach is to identify molecules that have multiple biological activities that are relevant to the disease. Fisetin is a small, orally active molecule which can act on many of the target pathways implicated in AD. We show here that oral administration of fisetin to APPswe/PS1dE9 double transgenic AD mice from 3 to 12 months of age prevents the development of learning and memory deficits. This correlates with an increase in ERK phosphorylation along with a decrease in protein carbonylation, a marker of oxidative stress. Importantly, fisetin also reduces the levels of the cyclin-dependent kinase 5 (Cdk5) activator p35 cleavage product, p25, in both control and AD brains. Elevated levels of p25 relative to p35 cause dysregulation of Cdk5 activity leading to neuroinflammation and neurodegeneration. These fisetin-dependent changes correlate with additional anti-inflammatory effects, including alterations in global eicosanoid synthesis, and the maintenance of markers of synaptic function in the AD mice. Together, these results suggest that fisetin may provide a new approach to the treatment of AD.

## Introduction

Although Alzheimer’s disease (AD) is defined in terms of plaque and tangle pathology, it is most frequently associated with other detrimental events such as microvascular damage and inflammation (Schubert & Maher, 2012). By far, the major risk factor for AD is old age (Herrup, 2010), and age-related decreases in cognitive function in both non-AD and AD subjects involve alterations in multiple cellular processes. Therefore, it is unlikely that hitting a single target will result in significant benefits to patients with AD (Frautschy & Cole, 2010). This conclusion has been largely borne out by the failure of numerous clinical trials for single target drugs in AD (Golde *et al*., 2011). Nevertheless, current drug research efforts continue to be almost exclusively focused on single protein targets and the identification of small molecules that can modulate these targets with high affinity (Schubert & Maher, 2012). An alternative approach is to identify small molecules that have multiple biological activities that are relevant to AD.

We have identified a small, orally active molecule, fisetin, which can act on many of the target pathways implicated in AD (Maher, 2009). Fisetin was originally identified in a screen for flavonoids that could prevent oxidative stress-induced nerve cell death (Ishige *et al*., 2001). Of the ~30 flavonoids tested in this study, fisetin was one of the most potent. Additional studies showed that fisetin also possessed neurotrophic activity, a property that distinguished it from the other flavonoids tested (Sagara *et al*., 2004). We then showed that fisetin could facilitate long-term potentiation in hippocampal slices in an ERK-dependent manner and that oral administration promoted memory in normal mice (Maher *et al*., 2006). More recently, we demonstrated that fisetin was effective at slowing death in multiple models of Huntington’s disease in a partially ERK-dependent manner (Maher *et al*., 2011). Fisetin also has anti-inflammatory activity both *in vitro* and *in vivo* (Gelderblom *et al*., 2012). Furthermore, it can reduce Aβ fibril formation *in vitro* (Kim *et al*., 2005). Together, these observations indicate that fisetin has multiple properties that together might be able to prevent the decline in brain function associated with AD.

As fisetin improves memory and has *in vivo* anti-inflammatory and *in vitro* neurotrophic and anti-amyloid properties, we investigated its effects in an AD disease model, the APPswe/PS1dE9 double transgenic AD mouse. The purpose of the study was to determine whether fisetin could reduce AD-associated learning and memory deficits and to see how these actions correlated with its effects on AD-related processes implicated in disease progression including amyloid burden, gliosis, oxidative damage, inflammatory pathways and their eicosanoid products as well as dysregulation of the cyclin-dependent kinase 5 (Cdk5) activator p35. The cleavage of p35 to p25 results in hyperactivation and dysregulation of Cdk5 activity, and the accumulation of p25 has been implicated as a causative factor in AD (Lopes *et al*., 2010). Importantly, p25 overexpression is associated with inflammation and astrogliosis along with synaptic damage (Muyllaert *et al*., 2008; Sundaram *et al*., 2012). Together, our results demonstrate that fisetin, a compound that activates multiple, well-defined neuroprotective pathways, may provide a new approach to the treatment of AD.

## Results

The APPswe/PS1dE9 double transgenic (AD) mice (Jankowsky *et al*., 2001) used for these studies show significant plaque deposition by 8–9 months of age (Jankowsky *et al*., 2005) as well as clear cognitive impairment (Jankowsky *et al*., 2005). Therefore, it was initially asked whether fisetin could reduce these changes. Two groups each of wild-type and AD mice were used for these studies. Fisetin was fed to one set of wild-type and one set of AD mice between the ages of 3 and 12 months in their food at 0.05%, resulting in a daily dose of approximately 25 mg kg^−1^. This dose of fisetin was chosen based on earlier studies on fisetin and cognitive function in mice (Maher *et al*., 2006).

**Figure 1:**
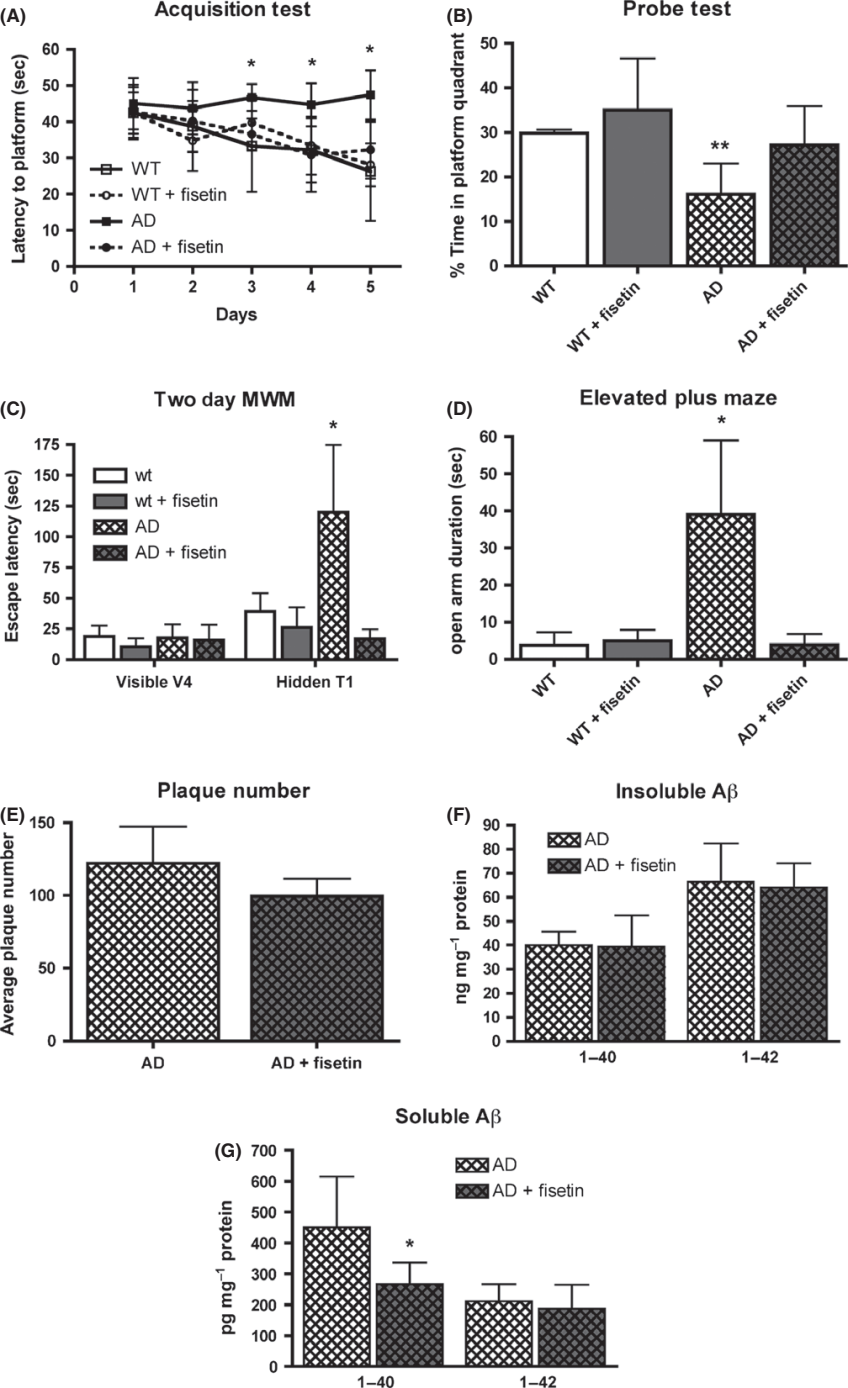
Fisetin maintains learning and memory in AD mice but does not affect plaque number. (A) Performance of the mice during the acquisition phase of the MWM test. Nine-month-old AD mice failed to learn the task over the 5-day testing period, whereas fisetin-treated AD mice learned to locate the submerged platform. (B) Performance of the mice during the memory phase of the MWM test. AD mice did not spend a greater than random percentage of their time in the quadrant where the submerged platform was previously located, whereas fisetin-treated AD mice spent greater than random amounts of time in the platform quadrant. (C) Performance of 12-month-old mice during the 2-day WM test. Although all the mice learned to find the visible platform on day 1, the AD mice did not remember where the platform was located when it was submerged on day 2, whereas fisetin-treated AD mice did remember. (D) 12-month-old AD mice spend more time in the open arm of the elevated plus maze, an indicator of disinhibition, as compared with fisetin-treated AD mice. Data represent means ± SD, *n* = 9–12 per group. Analysis by one-way anova followed by Tukey-Kramer multiple comparisons *post hoc* test. *P < 0.05 and **P < 0.01 relative to control non-Tg mice. (E) 30 micrometer thick coronal sections from 12-month-old AD mice were stained with antibody 6E10, and plaque counts in the hippocampus were quantified using Image J software. The average plaque counts for each mouse group is expressed as the number of plaques ± SD (*n* = 6 per group; unpaired *t*-test). Aβ_1-40_ and Aβ_1-42_ levels were measured in the insoluble (100 000 *g* pellet) (F) and RIPA soluble (G) hippocampal fractions by ELISA in untreated AD mice and AD mice fed fisetin. The results are expressed as mean ± SD, *n* = 6–8 per group. Analysis by unpaired *t*-test. *P < 0.05. AD, Alzheimer’s disease; MWM, Morris water maze.

We tested the performance in both the learning and memory arms of the Morris water maze (MWM) at 9 months of age and then repeated the memory arm in a 2-day version of the water maze (WM) (Gulinello *et al*., 2009) at 12 months. At 9 months of age, wild-type mice showed decreased times to find the hidden platform over the 5 days of the MWM assay (Fig. 1A). Fisetin had no significant effect on the behavior of the wild-type mice in the acquisition task. In contrast, the AD mice showed very little learning over the 5 days of the acquisition task, while the fisetin-fed AD mice behaved almost indistinguishably from the wild-type mice in the acquisition task (Fig. 1A). In the probe test (Fig. 1B), which specifically assays memory, the wild-type mice and the wild-type mice fed fisetin both showed significant recall of the platform quadrant spending >25% of their time in this quadrant. In contrast, the AD mice showed no indication of memory, spending only 16% of their time in the platform quadrant. As the platform quadrant is on the opposite side of the tank from where the mice enter, it is not surprising that they spend <25% of their time in this quadrant. However, the AD mice fed fisetin showed clear evidence of memory improvement. Fisetin had no effect on swim speeds (not shown).

As AD is a progressive disease, we tested the mice again at 12 months to determine whether fisetin continued to reduce memory deficits. As we knew from previous studies that we could not repeat the MWM on the same set of mice, we used an alternative version that only tests memory (Gulinello *et al*., 2009) and addresses some of the confounding factors that arise when testing older mice in the MWM. In the 2 day WM, on day 1, mice are trained to find a visible platform using four trials. All of our mice, regardless of genotype or diet, were able to find the visible platform within 30 s by the last visible trial (Fig. 1C). This result indicated that neither the genotype nor the diet affected the animals’ ability to swim or to locate the visible platform. Thus, any impairment in spatial memory seen with the 2 day WM will reflect a cognitive deficit and not a lack of motivation or visual function. 24 h after the final visible platform trial, the mice were tested in the same tank but with the platform hidden. As shown in Fig. 1C, the escape latency of the AD mice was significantly longer than that of the wild-type mice, similar to what was reported for triple transgenic AD mice (Gulinello *et al*., 2009). In contrast, the escape latency for the AD mice fed fisetin was indistinguishable from that for the wild-type mice.

Another characteristic of AD is social disinhibition which can result in inappropriate behavior in patients with AD. The elevated plus maze (EPM), a rodent model of anxiety, is often used as a measure of disinhibition in mice (Walf & Frye, 2007). The AD mice used in this study were previously shown to exhibit increased time in the open arms in the EPM (Pugh *et al*., 2007), which was interpreted as indicative of disinhibition. As shown in Fig. 1D, we also found that the AD mice spent significantly more time in the open arm than the wild-type mice. This behavior was reversed in the mice fed fisetin.

Given the clear improvement in both cognitive and neuropsychiatric behavior in the AD mice fed fisetin, we next asked what biochemical changes might underlie these effects of fisetin.

As the AD mice are characterized by increases in amyloid-β (Aβ plaques), we first looked at plaque loads in sections of brains from these mice. No significant differences in Aβ plaque loads were seen (Fig. 1E). We also looked at Aβ levels in the radioimmunoprecipitation assay buffer (RIPA) insoluble (100 000 *g* pellet) and soluble (RIPA supernatant) fractions of the hippocampi of fisetin-fed and control AD mice. Neither Aβ_1-40_ nor Aβ_1-42_ levels, as measured by ELISA, were altered in the RIPA insoluble fraction in the animals fed fisetin relative to untreated animals (Fig. 1F). However, fisetin treatment did significantly reduce the levels of Aβ_1-40_ but not Aβ_1-42_ in the RIPA soluble fraction (Fig. 1G).

**Figure 2:**
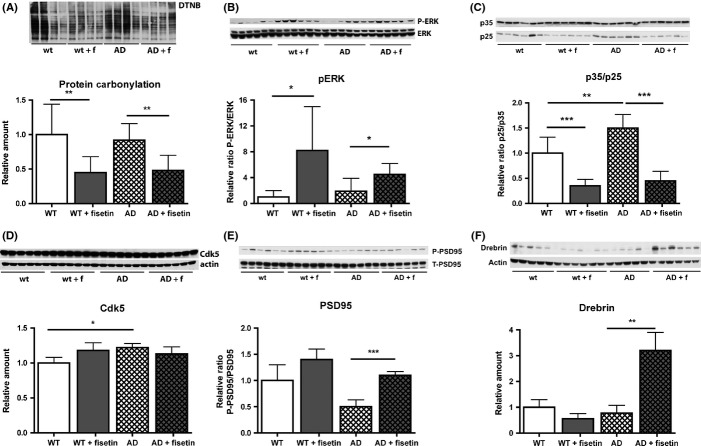
Fisetin reduces oxidative stress, increases ERK activation, and maintains p35. (A) Fisetin reduces protein carbonylation, a marker of oxidative stress, in hippocampal extracts of non-Tg and AD mice. (B) Fisetin increases ERK activation in non-Tg and AD mice. (C) Fisetin reduces the cleavage of the Cdk5 activator p35 to pro-neurodegeneration p25. (D) Fisetin does not alter Cdk5 levels. (E) The phosphorylation of the p35 substrate PSD95, which correlates with synapse efficacy, is reduced in AD mice and maintained by fisetin. (F) Fisetin increases the synaptic spine marker drebrin in AD mice. Representative Western blots are shown. Data represent means ± SD, *n* = 9–12 per group. Analysis by one-way anova followed by Tukey-Kramer multiple comparisons *post hoc* test. *P < 0.05, **P < 0.01 and ***P < 0.001 relative to control non-Tg mice or AD mice. AD, Alzheimer’s disease; PSD95, postsynaptic density protein 95; Cdk5, cyclin-dependent kinase 5.

Previously, it was shown that fisetin is able to reduce markers of oxidative stress in cell culture neuroprotection assays (Ishige *et al*., 2001) and to induce ERK phosphorylation in cultured cells (Sagara *et al*., 2004) and mouse hippocampal slices (Maher *et al*., 2006). Both increases in oxidative stress (Sonnen *et al*., 2008) and decreases in ERK-dependent signaling (Giovannini *et al*., 2008) have been implicated in decreases in cognitive function in AD. Furthermore, ERK activation plays a key role in synaptic plasticity and memory (Sweatt, 2004). Thus, we first looked at fisetin’s effects on these parameters. As shown in Fig. 2A, fisetin reduced oxidative stress in both control and AD mice as determined by changes in protein carbonylation using the OxyBlot kit. Fisetin also increased ERK phosphorylation both in wild-type and AD mice (Fig. 2B).

ERK activation induces the expression of p35, a neuron-specific activator of Cdk5 (Desbarats *et al*., 2003). Cleavage of p35 to its truncated form, p25, which results in the aberrant activation of Cdk5, has been linked to disruptions in synaptic plasticity and integrity in AD (Cruz & Tsai, 2004). While Cdk5 activation by p35 is associated with its normal physiological activities, activation by the truncated form, p25, abnormally stimulates Cdk5 activity. As ERK phosphorylation was altered by fisetin, the effect of fisetin and AD on the levels of p35 and p25 in the mouse hippocampus was assayed. While fisetin did not significantly alter p35 levels in the hippocampi of the AD mice, it did prevent the highly significant increase in p25 levels in AD mice and greatly reduced the p25/p35 ratio in both wild-type and AD mice (Fig. 2C). Fisetin did not alter the levels of Cdk5 in any mice (Fig. 2D). The Cdk5/p35 complex modulates the activity of a variety of proteins, including synapsin 1 and postsynaptic density protein 95 (PSD95), through phosphorylation. As shown in Fig. 2E, PSD95 phosphorylation was reduced in the AD brains and this was restored by fisetin. Fisetin also restored the levels of the PSD95-associated protein drebrin (Fig. 2F). An elevation of drebrin above control levels was also seen with the neurotrophic compound J147 (Chen *et al*., 2011).

**Figure 3:**
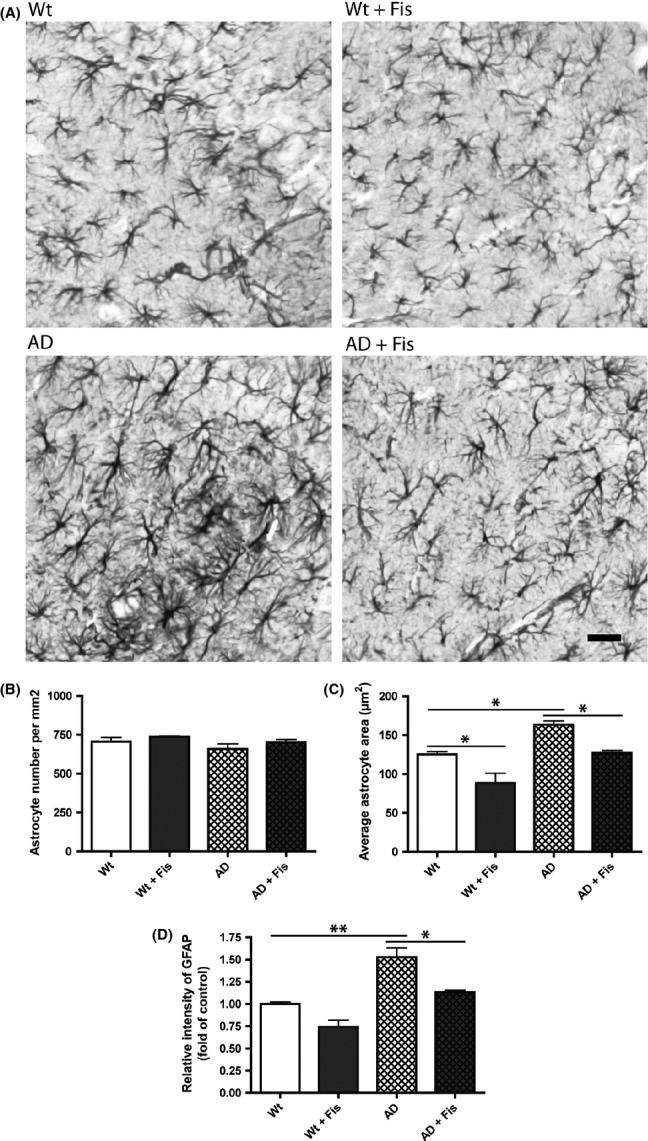
Fisetin reduces astrocytic reactivity in the brains of AD mice. (A) Representative images of GFAP immunostaining in the hippocampus. The graphs show quantification of (B) the number of astrocytes per mm^2^, (C) the average astrocyte area (μm^2^), and (D) the intensity of GFAP staining in relation to non-Tg mice. Scale bar: 10 μm. Data represent means ± SEM, *n* = 3 per group (4–5 slices per brain). Analysis by one-way anova followed by Tukey-Kramer multiple comparisons *post hoc* test. *P < 0.05 and **P < 0.01 relative to control non-Tg mice or AD mice. GFAP, glial fibrillary acidic protein.

**Figure 4:**
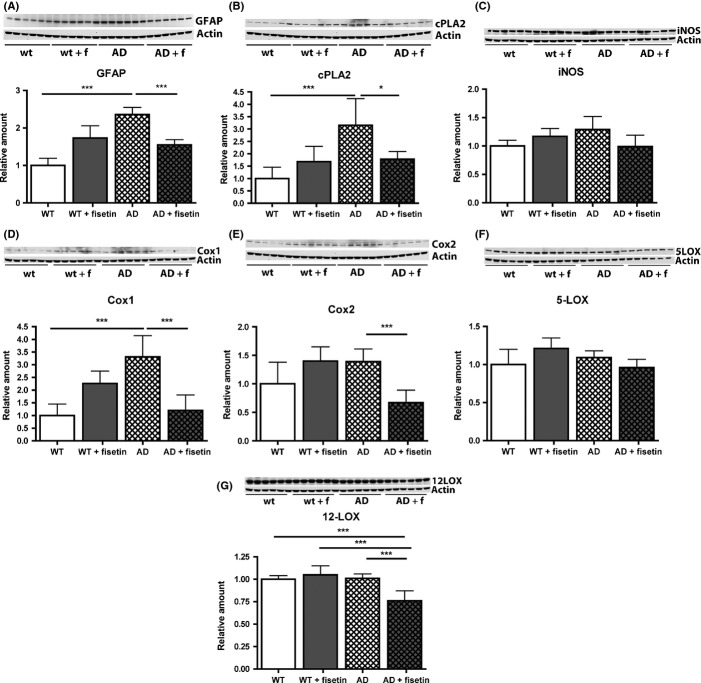
Fisetin reduces the protein expression of markers of inflammation in the AD mice. (A) GFAP, (B), PLA2c, (C), iNOS (D), Cox1 (E) Cox2, (F) 5-LOX and (G) 12-LOX. Representative Western blots are shown. Data represent means ± SD, *n* = 9–12 per group. Analysis by one-way anova followed by Tukey-Kramer multiple comparisons *post hoc* test. *P < 0.05 and ***P < 0.001 relative to control non-Tg mice or AD mice. AD, Alzheimer’s disease; GFAP, glial fibrillary acidic protein; iNOS, inducible nitric oxide synthase; Cox1, cyclooxygenase 1.

Recently, using mice overexpressing p25 specifically in neurons, it was shown that p25 induces neuroinflammation that is associated with astrogliosis (Muyllaert *et al*., 2008; Sundaram *et al*., 2012). Thus, given the effects of fisetin on p25 levels in the AD mice, we looked at glial fibrillary acidic protein (GFAP) levels, a marker of astrogliosis, in both wild-type and AD mice in the absence and presence of fisetin treatment. As shown in Fig. 3, while AD had no effect on the number of astrocytes in the hippocampus (Fig. 3B), it significantly increased both their area and the intensity of GFAP staining (Fig. 3C,D). This alteration was largely reversed by fisetin treatment. A fisetin-dependent reduction in GFAP levels was also seen in the hippocampus of the AD mice by Western blotting (Fig. 4A). Upregulation of cytosolic phospholipase A2 (cPLA2) was found to play a key role in the astrogliosis seen in p25-overexpressing mice (Sundaram *et al*., 2012). Consistent with this observation, cPLA2 levels were significantly increased in the hippocampi of AD mice but were restored to control values by fisetin treatment (Fig. 4B). Cyclooxygenase 1 (Cox1) was also increased in the AD mice (Fig. 4D) and this protein along with cyclooxygenase 2 (Cox2) and 12-lipoxygenase (12-LOX) was reduced by treatment with fisetin (Fig. 4E and G). As Cox1 is constitutively expressed, the increase in Cox1 levels in the AD mice may be due to an increase in the number of Cox1-producing cells such as microglia. The levels of inducible nitric oxide synthase (iNOS) and 5-lipoxygenase (5-LOX) were not statistically different between the groups (Fig. 4C and F).

**Figure 5:**
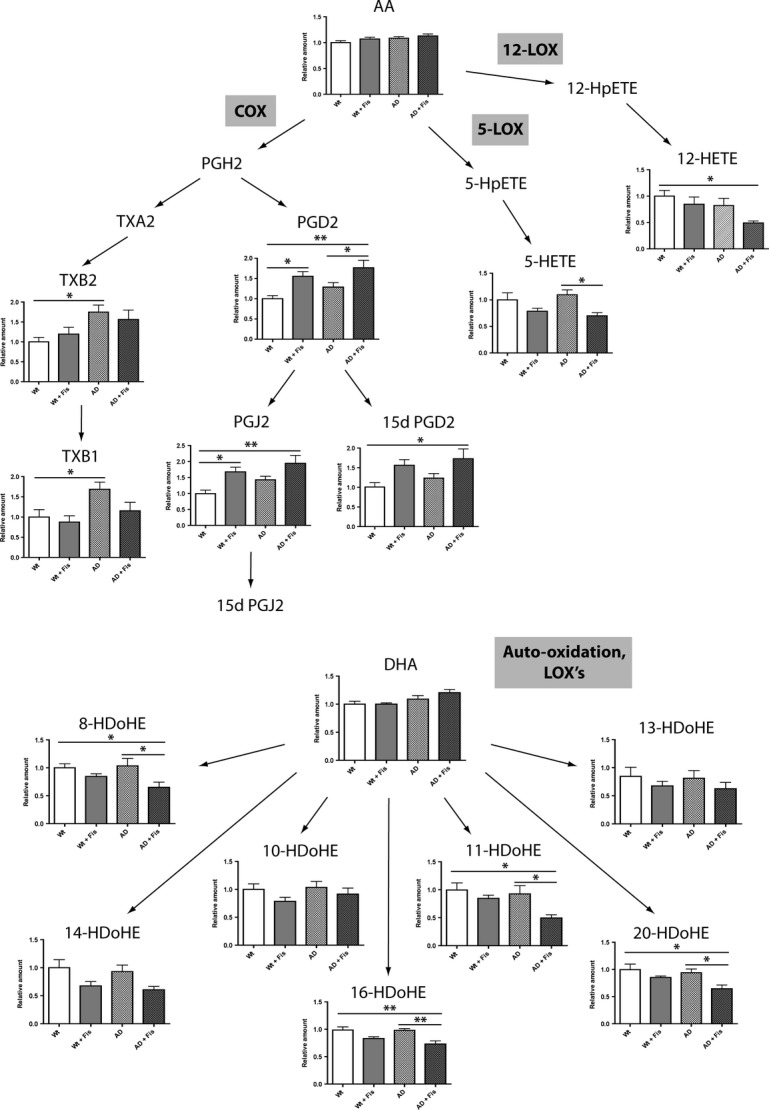
Fisetin modulates the metabolism of AA and DHA eicosanoid derivatives in AD mice. Data represent means ± SEM, *n* = 6 per group. Analysis by one-way anova followed by Newman–Keuls *post hoc* test. *P < 0.05 and **P < 0.01 relative to control non-Tg mice or AD mice. AD, Alzheimer’s disease.

To further elucidate the effects of fisetin on inflammation, we conducted a detailed analysis of eicosanoid production in the wild-type and AD mice with and without fisetin treatment using liquid chromatography tandem mass spectrometry (Fig. 5). This approach generated global profiles consisting of over 160 individual eicosanoids many of which were altered in AD and after fisetin treatment. Eicosanoids are a class of bioactive lipid mediators derived from the metabolism of polyunsaturated fatty acids, namely arachidonic acid (AA), by COXs, LOXs, cytochrome P450s, and nonenzymatic pathways (Buczynski *et al*., 2009). They are known to be potent endogenous regulators of the inflammatory response in the periphery but are much less well studied in the brain. We found that AD increased the production of the pro-inflammatory thromboxanes TXB1 and TXB2 and this was partly prevented by fisetin. On the other hand, fisetin increased production of prostaglandin D2 (PGD_2_) and its nonenzymatic anti-inflammatory products prostaglandin J2 (PGJ_2_) and 15-deoxy-PGD_2_ (15d-PGD_2_). The effects of AD and fisetin on LOX metabolites are also shown in Fig. 5. While AD did not have any significant effect on the levels of LOX metabolites, fisetin significantly reduced the levels of pro-inflammatory 5-hydroxyeicosatetraenoic acid (5-HETE) and 12-hydroxyeicosatetraenoic acid (12-HETE), the primary metabolites of 5-LOX and 12-LOX, respectively, in the AD mice. Furthermore, this analysis showed that fisetin strongly reduced the levels of multiple monohydroxydocosahexaenoic acids (HDoHE), metabolic derivatives of docosahexaenoic acid (DHA), in the AD mice. These can be generated by either auto-oxidation of DHA or enzymatic metabolism of DHA via LOX pathways (Reynaud *et al*., 1993).

Eicosanoids are both products and regulators of the innate immune system whose dysregulation has been implicated in AD (for review see Butchart & Holmes, 2012). Therefore, we looked at the RNA expression of components of the innate immune system in the brains of our mice and found that several members of the complement and the toll-like receptor (TLR) pathways were significantly upregulated in the AD mice (Table 1). While fisetin did not affect the expression of any of the complement markers, it did prevent the increase in CD40 and lowered the expression of some of the TLRs that were elevated in the AD mice.

**Table 1:** RNA quantitative analysis showing that the complement and the TLR inflammatory pathways are activated in the AD mice

	Wt	Wt+Fis	AD	AD + Fis
C1qa	2000.56 ± 138.3	2026.48 ± 115.42	4041.51 ± 477.58*	3522.79 ± 435.1*
C1qb	3172.4 ± 195.47	3868.31 ± 253.55	7441.85 ± 273.81***	7475 ± 571.93***
C1r	321.68 ± 4.15	362.45 ± 35.88	343.16 ± 12.46	340.72 ± 12.63
C1s	52.82 ± 3.22	53.57 ± 9.15	64.68 ± 3.83	60.42 ± 5.5
C2	103.61 ± 3.37	102.92 ± 7.41	105.55 ± 14.41	118.12 ± 3.99
C3	N.d.	N.d.	N.d.	N.d.
C3ar1	142.47 ± 7.12	153.09 ± 16.95	416.54 ± 35.83***	411.6 ± 38.6***
C4a	821.5 ± 55.47	972.85 ± 218.94	3018.03 ± 210.14***	2792.64 ± 195.29***
C6	N.d.	N.d.	N.d.	N.d.
C7	N.d.	N.d.	N.d.	N.d.
C8a	N.d.	N.d.	N.d.	N.d.
C8b	N.d.	N.d.	N.d.	N.d.
C9	N.d.	N.d.	N.d.	N.d.
Cfb	33.33 ± 5.15	35.18 ± 8.77	64.56 ± 4.52*	63.2 ± 2.76*
Cfd	N.d.	N.d.	N.d.	N.d.
Cd55	220.24 ± 12.18	174.02 ± 0.85*	161.03 ± 19.94*	169.83 ± 7.77*
Tlr1	14.96 ± 3.41	15.94 ± 2.89	35.68 ± 7	36.94 ± 10.01
Tlr2	47.87 ± 3.83	41.91 ± 2.59	83.2 ± 7.96**	71.15 ± 5.97*
Tlr3	517.17 ± 8.06	509.25 ± 26.77	538.37 ± 9.86	534.04 ± 24.49
Tlr4	86.89 ± 3.85	88.58 ± 8.63	105.37 ± 2.22	103.25 ± 9.07
Tlr5	N.d.	N.d.	N.d.	N.d.
Tlr6	84.89 ± 17.99	81.33 ± 14.43	87.43 ± 11.41	130.68 ± 12.47
Tlr7	100.48 ± 6.93	94.82 ± 8.1	186.99 ± 18.57**	163.26 ± 16.82*
Myd88	90.8 ± 2.48	112.52 ± 6.82	140.61 ± 9.31**	131.9 ± 9.69*
Nfkb1	514.78 ± 13.91	507.57 ± 18.96	539.09 ± 8.79	523.12 ± 11.26
Nos2	14 ± 1.8	15.23 ± 3.64	25.09 ± 2.95	18.88 ± 3.46
Tgfb1	320.62 ± 28.64	282.07 ± 22.95	478.88 ± 33.67**	460.92 ± 25.97**
Tgfb2	527.99 ± 27.72	490.25 ± 20.36	498.92 ± 55.55	485.01 ± 27.39
Tgfb3	481.06 ± 19.57	430.96 ± 23.11	459.16 ± 5.89	408.42 ± 10.89
Tgfbr1	1130.43 ± 25.59	984.71 ± 24.14**	1295.7 ± 45.64**	1308.75 ± 11.82**
Cd40	16.71 ± 2.52	24.97 ± 0.99	34.93 ± 2.87**	25.22 ± 2.32[Table-fn tf1-2]
Cd401g	N.d.	N.d.	N.d.	N.d.

Data represent means ± SEM, *n* = 3 per group. Analysis by one-way anova followed by Newman–Keuls *post hoc* test.

*P < 0.05, **P < 0.01, ***P < 0.001 relative to control non-Tg mice or ^‡^*P* < 0.05 relative to AD mice.

N.d., Not detected; TLR, toll-like receptor; AD, Alzheimer’s disease.

We also evaluated the effects of long-term feeding of fisetin on the health of the animals. No significant differences in body weights were seen between the groups. Following sacrifice, multiple tissues (lungs, spleen, liver, kidneys, heart, stomach, intestine, testes, and ovary) were examined using standard toxicological pathology criteria and no toxicity was associated with fisetin treatment. Furthermore, fisetin showed no toxicity at doses up to 2 g kg^−1^ in an acute toxicity assay and was negative in the Ames test.

## Discussion

**Figure 6:**
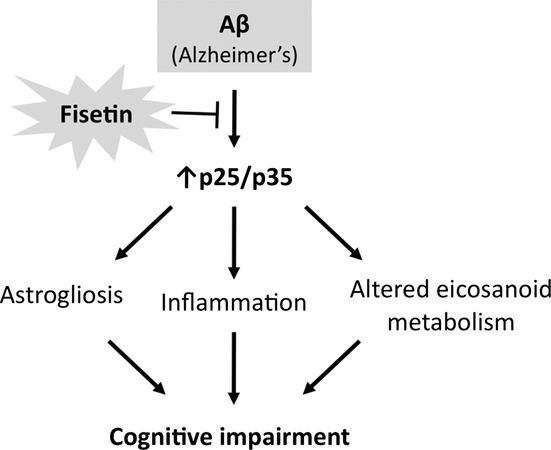
Summary of the protective mode of action of fisetin in the AD mice. AD, Alzheimer’s disease.

The above data show that oral administration of the flavonoid fisetin prevents the cognitive and neuropsychiatric problems that develop as a consequence of AD. A major nexus for the effects of fisetin in the AD mice appears to be the Cdk5 activator p35 (Fig. 6). Importantly, fisetin can induce ERK activation both *in vitro* (Sagara *et al*., 2004; Maher *et al*., 2006) and, as shown in Fig. 2, in mice and ERK activation can induce p35 expression (Desbarats *et al*., 2003). Furthermore, in cells, both GSH and oxidized GSH (GSSG) were found to inhibit the activity of calpain (Rackoff *et al*., 2001), the protease implicated in the cleavage of p35 to p25. Fisetin is able to maintain GSH levels under conditions of oxidative stress (Ishige *et al*., 2001). Thus, fisetin may be particularly effective at maintaining p35 because of its ability to both stimulate ERK activity as well as to reduce p35 cleavage. This in turn may contribute to the maintenance of markers of synaptic function such as PSD95 and drebrin and the decrease in markers of neuroinflammation. Although another of the consequences of p35 cleavage in AD can be an induction of tau phosphorylation (Lopes *et al*., 2010), no increases in tau phosphorylation were seen in the AD mice in our hands (not shown), so it was not possible to evaluate the effects of fisetin on this parameter.

Consistent with a recent study on the consequences of p25-overexpression in mice (Sundaram *et al*., 2012), astrogliosis was seen in the AD mouse brains. It was suggested that this was dependent on the p25-mediated activation of cPLA2. We detected an increase in cPLA2 levels in the AD mice which, along with astrogliosis, was prevented by fisetin treatment. cPLA2 levels were also shown to be increased in human AD brains along with other markers of inflammation including Cox2 (Rao *et al*., 2011). Furthermore, both astrocytes and microglia have been reported to be activated in AD and their activation is implicated in the loss of nerve cell function (Rao *et al*., 2011). In particular, activation of astrocytes results not only in the production of pro-inflammatory cytokines but also in a decrease in their normal role as guardians of nerve cell function (Steele & Robinson, 2012).

The other key finding from this study is that both AD and fisetin alter eicosanoid biosynthesis in the brain with the overall result being a reduction in pro-inflammatory products by fisetin. TXB_2_ and TXB_1_ were found significantly elevated in the AD brains and were partly reduced by fisetin. The thromboxane pathway is implicated in platelet aggregation, adhesion, and vascular contraction during inflammation (Hardwick *et al*., 2013). This is of relevance given the prothrombotic effect of TXs and the link between thrombosis and vascular pathology in AD (Cortes-Canteli *et al*., 2010). Thus, fisetin has anti-inflammatory effects at multiple levels.

The increased production of PGD_2_ and its products PGJ_2_ and 15d-PGD_2_ by fisetin is of particular interest because these prostaglandins have potent anti-inflammatory effects in brain microglia (Yoon *et al*., 2008) including down regulation of iNOS, a known effect of fisetin (Gelderblom *et al*., 2012). Historically, PGD_2_ was regarded as a pro-inflammatory mediator but a potentially anti-inflammatory role is now also recognized (Scher & Pillinger, 2009). It is unclear what determines PGD_2_’s inflammatory status, but our data showing an improvement in cognitive performance and a reduction in the expression levels of pro-inflammatory markers in the AD mice by fisetin suggest that the increased levels of PGD_2_ and its metabolites might be associated with the anti-inflammatory action of fisetin in the brain. Furthermore, PGD_2_ was reported to stimulate ERK activation (Choi *et al*., 2011), which is in accordance with the enhanced ERK phosphorylation seen in both Wt and AD mice treated with fisetin. Whether ERK activation can, in turn, modulate the production of PGD_2_ and its metabolites remains to be addressed. Importantly, hematopoietic PGD synthase (HPGDS), a member of σ-class of glutathione-S-transferases, has the ability to convert PGH_2_ into PGD_2_ in the presence of GSH (Hardwick *et al*., 2013). Given that fisetin is able to maintain GSH levels under conditions of oxidative stress (Ishige *et al*., 2001), it might be that the increase in PGD_2_ and its derivatives in mice treated with fisetin is in part mediated by the maintenance of GSH levels in the brain, thereby affecting the activity of PGD synthase. In contrast with lipocalin PGD synthase, which also converts PGH_2_ into PGD_2_ but is GSH-independent (Hardwick *et al*., 2013), HPGDS is expressed in immune and inflammatory cells. Importantly, HPGDS is upregulated in microglia and astrocytes surrounding Aβ plaques in human patients with AD and in an AD mouse model (Mohri *et al*., 2007).

Fisetin has previously been shown to inhibit 5, 12 and 15-LOXs in cell-based assays (for review see Maher, 2009). However, whether it could also do so *in vivo* was not known. Both 5-LOX and 12-LOX metabolites induce activation of NF-κB and the expression of pro-inflammatory cytokines (Hardwick *et al*., 2013). The results shown in Fig. 5 in combination with the protein expression data shown in Fig. 4 clearly indicate that fisetin is able to inhibit both 5-LOX and 12-LOX *in vivo*, at least in the context of AD.

HDoHEs are metabolic derivatives of DHA generated by either auto-oxidation of DHA or enzymatic metabolism of DHA via the LOX pathways (Reynaud *et al*., 1993). Fisetin had a potent inhibitory effect on several of the HDoHEs, which is in accordance with the anti-oxidative potential of fisetin and its inhibitory properties on the LOXs.

Analysis of RNA expression showed that both the complement and TLR pathways were activated in the AD mice. This is of particular relevance given that the complement system is also activated in human patients with AD but that it is thought to be part of a neuroprotective response that helps clear apoptotic cells and Aβ peptide (Benoit *et al*., 2013). Therefore, there is a component of the innate immune system that must remain turned on and is beneficial in the context of AD. The fact that fisetin did not alter this system could be of therapeutic significance. Similarly, the relevance of some TLRs to the clearance of Aβ from the brain of AD mouse models has been demonstrated (Landreth & Reed-Geaghan, 2009). Specifically, TLR2 and TLR4 have been shown to mediate the activation of microglia which take up and clear Aβ from the brain (Landreth & Reed-Geaghan, 2009). However, the innate immune system is a double-edged sword and if a persistent inflammatory response exists, as in patients with AD, it may contribute to and exacerbate the pathology. In this context, activation of TLR4 robustly stimulates eicosanoid biosynthetic pathways, thereby further contributing to local inflammation (Quehenberger & Dennis, 2011). Our data show the upregulation of several TLRs in the AD mice. Interestingly, although not statistically significant, fisetin appears to lower the levels of these TLRs. Fisetin also significantly prevented the AD-dependent increase in CD40 which is known to potentiate TLR signaling and is found associated with AD (Giunta *et al*., 2010).

Fisetin was also able to reduce protein carbonylation, a marker of oxidative stress. Increases in oxidative stress have long been associated with both mouse models and human AD (Sonnen *et al*., 2008) but the exact mechanisms underlying this increase are still not clear. The results presented here suggest that the increases in oxidative stress are more likely to be associated with the increases in neuroinflammation rather than Αβ levels as while fisetin had no effect on Aβ plaque load and only a modest effect on soluble Aβ_1-40_ levels, it significantly reduced multiple markers of neuroinflammation in the AD mice. A key role for neuroinflammation in cognitive dysfunction in AD mice is supported by previous studies (Wirths *et al*., 2010).

Several recent epidemiological studies have provided evidence that higher consumption of dietary flavonoids and especially flavonols, the group to which fisetin belongs, is associated with lower rates of dementia (Beking & Vieira, 2010; Kesse-Guyot *et al*., 2011). These studies lend support to our results with fisetin in AD mice reported here.

Although p25 and the aberrant activation of Cdk5 have been suggested as therapeutic targets for the treatment of AD, it has not been clear how to achieve this. Inhibitors of Cdk5 were recently found to increase the levels of BACE1, the enzyme that initiates production of Aβ, suggesting that these compounds may not be useful for the treatment of AD (Sadleir & Vassar, 2012). In contrast, fisetin is a natural product that is clearly able to prevent the generation of p25 in the brains of AD mice and therefore has the potential to do so in humans. Furthermore, fisetin has additional properties including anti-inflammatory activity and the ability to reduce oxidative stress which occurs in all neurodegenerative diseases. In addition, as shown here, fisetin can modulate eicosanoid production promoting an overall anti-inflammatory profile. Together, these properties, and the fact that it is an apparently safe natural product, make fisetin a particularly attractive compound for the treatment of AD. Finally, our results suggest that analysis of eicosanoid profiles might provide a powerful tool in the future when assessing disease progression in disease models and human patients with AD.

## Experimental procedures

### huAPPswe/PS1ΔE9 transgenic mice

The AD transgenic mice (line 85; a generous gift of Dr. J.L. Jankowsky) have been previously characterized (Jankowsky *et al*., 2001). These mice carry two transgenes, the mouse/human chimeric *APP/Swe*, linked to Swedish FAD and human *PS1∆E9*. At 3 months of age, male transgenic mice and their wild-type littermates were fed a high fat diet (Harlan Teklad, Madison, WI, USA) with and without fisetin (0.05%). Food consumption was measured weekly, and there were no significant differences between the groups. These studies were approved by the Salk Institute IACUC.

### Behavioral assays

#### Morris water maze

Spatial learning and memory were determined in 9-month-old AD transgenic mice fed fisetin for the previous 6 months using the Morris water maze (Vorhees & Williams, 2006). The MWM procedure was four trials per day for five consecutive days. For each trial, mice were placed in the pool (water temperature = 27 °C) at one of four start locations. The starting locations were separated by 90° and mice started a trial once from each of the four possible start locations on each day. The latency to find and mount the hidden platform was used as the primary dependent variable. Swimming speeds were recorded to assess fisetin-induced motor effects. Mice were given a maximum of 50 s to find the hidden platform. If the mice failed to find the platform after 50 s, they were placed on the platform by the experimenter. All mice remained on the platform for 15 s before being placed in a heated incubator (30 °C) between trials. Personnel evaluating animals in the MWM were blinded to the genotype and diet of each animal. A mean daily latency to find the goal platform during MWM testing was computed for each mouse. On day 6, a 60-s probe test was performed in which the platform was removed and the time spent in the quadrant where the platform was previously located was determined. Mice were disqualified if they exhibited excessive floating or thigmotaxis.

#### Two day water maze

Spatial memory was determined in 12-month-old AD transgenic mice fed fisetin for 9 months using the 2-day water maze adapted from (Gulinello *et al*., 2009). The setup was the same as for the MWM. On day 1 of the 2 day WM procedure, the mice were trained to find a visible platform that remained in the same location for the four trials using cues located around the pool within a 180-s time frame. There were four visible platform trials (V1–V4) where the last visible platform trial of a mouse was considered the baseline. Mice were disqualified if they failed to reach the platform within 30 s in V4. On day 2, 24 h following the last visible platform trial, the mice were tested in a hidden platform trial for up to 180 s. The time it took for each mouse to find the hidden platform was measured as escape latency. Swimming speeds were recorded to assess fisetin-induced motor effects. All trials were recorded using the EthoVision software (Noldus, Leesburg, VA, USA), and statistics were computed using GraphPad Instat software (GraphPad, La Jolla, CA, USA).

#### Elevated plus maze

The EPM analyzes the anxiety response of mice and relies upon the tendency of mice to have a fear of heights and to navigate toward dark enclosed spaces and remain there (Walf & Frye, 2007). Mice are habituated to the room 24 h before testing. Mice are also habituated to the maze for 2 min before testing by placing them in the center of the maze and blocking entry to the arms. Mice are then tested in the maze for a 5-min period and a video-tracking system (Noldus EthoVision) tracks and records the behavior of the mice. Anxiety of mice is measured by comparing the time spent on the open arms to time spent on the closed arms. Statistics were computed using GraphPad Instat software.

### Tissue preparation and immunoblotting

Hippocampal tissue samples were homogenized in 10 volumes of RIPA lysis buffer (50 mm Tris, pH 7.5, 150 mm NaCl, 0.1% sodium dodecyl sulfate, 0.5% deoxycholate, and 1% NP40) containing a cocktail of protease and phosphatase inhibitors. Samples were sonicated (2 × 10 s) and centrifuged at 100 000 *g* for 60 min at 4 °C. Protein concentrations in the cell extracts were determined using the BCA protein assay (Pierce, Rockford, IL, USA). For SDS-PAGE, 20 μg of protein was used. All samples were separated using 10 or 12% Criterion XT Precast Bis-Tris Gels (Bio-Rad, Hercules, CA, USA). Proteins were transferred to nitrocellulose membranes and the quality of protein measurement, electrophoresis and transfer checked by staining with Ponceau S (Sigma-Aldrich, St. Louis, MO, USA). Membranes were blocked with 5% skim milk in TBS-T (20 mm Tris buffer pH 7.5, 0.5 m NaCl, 0.1% Tween 20) for 1 h at room temperature and incubated overnight at 4 °C in the primary antibody diluted in 5% BSA in TBS/0.05% Tween 20. The primary antibodies used were mouse anti-phospho-ERK (#9106, 1/1000), rabbit anti-ERK (#9102, 1/1000), rabbit anti-cPLA2 (#5249, 1/1000), rabbit anti-Cox1 (#4841, 1/1000), rabbit anti-Cox2 (#4842, 1/1000), rabbit anti-p35/25 (#2680, 1/1000), rabbit anti-phospho (tyr340)-PSD95 (#2930, 1/1000), rabbit anti-PSD-95 (#2507, 1/1000) and HRP-conjugated rabbit anti-actin (#5125, 1/20,000) from Cell Signaling (Beverly, MA, USA); rabbit anti-GFAP (#AD5804, 1/5000) from Millipore (Billerica, MA, USA); mouse anti-iNOS (#610431, 1/1000) and mouse anti-5-LOX (#610694, 1/1000) from BD Biosciences (San Jose, CA, USA); rabbit anti-12-LOX (#160304, 1/1000) from Cayman (Ann Arbor, MI, USA) and mouse antidrebrin (#ab12350, 1/1000) from Abcam (Cambridge, UK). Protein oxidation was assessed using the OxyBlot kit (Millipore) according to the manufacturer’s instructions. Subsequently, blots were washed in TBS/0.05% Tween 20 and incubated for 1 h at room temperature in horseradish peroxidase-goat anti-rabbit or goat anti-mouse (Bio-Rad) diluted 1/5000 in 5% skim milk in TBS/0.1% Tween 20. After additional washing, protein bands were detected by chemiluminescence using the SuperSignal West Pico Substrate (Pierce). For all antibodies, the same membrane was reprobed for actin or an antiserum reacting with the total protein. Autoradiographs were scanned using a Bio-Rad GS800 scanner. Band density was measured using the manufacturer’s software. Proteins were normalized to actin band density. Phosphoprotein levels were normalized to total protein expression. anova with the Tukey’s *post hoc* test was used to determine differences between means for Western blot analysis.

### Immunohistochemistry

Brains were fixed with 4% paraformaldehyde in 100 mm sodium tetraborate, pH 9.5, for 3 h, cryoprotected with 20% sucrose-potassium-PBS (KPBS), and cryostat sectioned into coronal (30 μm) sections. Sections were submerged in 0.3% H_2_O_2_ for 10 min to eliminate endogenous peroxidase activity and treated with 1% borate to eliminate free paraformaldehyde. Sections were incubated with primary antibody in 0.3% Triton X-100 in KPBS plus 2% filtered serum or BSA overnight at 4 °C, and with primary antibodies (1:1000) in 0.3% Triton X-100 for 1 hr at room temperature. After incubation with secondary antibody and ABC reagent (Vector Laboratories, Burlingame, CA, USA), sections were developed using metal-enhanced DAB solution. Sections were mounted to slides, dried, dehydrolyzed, treated with xylene, and covered using dibutyl phthalate xylene. Images were captured by a Zeiss digital camera connected to a Zeiss VivaTome microscope, and image analysis on sections was performed using Axiovision software.

Quantification of amyloid plaque burden was based on the image captured by immunohistochemical staining with antibody 6E10. Sections of each mouse cortex and hippocampus were imaged together, and the areas and densities of the plaques in the hippocampus only were measured by the Image J software (NIH, Bethesda, MD, USA). The total counts of Aβ plaques in sections per six mouse brains of each group were determined in an unbiased fashion.

Quantification of astrogliosis was based on the image captured by immunohistochemical staining with GFAP antibody. Sections of each mouse hippocampus were imaged together and the number of astrocytes per mm^2^, average astrocyte area per μm^2^, and relative intensity of GFAP staining were measured by the Image J software (NIH). Total counts in 4–5 sections per three mouse brains of each group were determined in an unbiased fashion.

### Aβ ELISA

Aβ 1-40 and 1-42 levels in hippocampal lysates were analyzed using the Aβ_1-40_ and Aβ_1-42_ ELISA kits from Invitrogen (# KHB3481 and # KHB3442, respectively; Carlsbad, CA, USA) according to the manufacturer’s instructions.

### Eicosanoid analysis

#### Eicosanoid extraction

All solvents were of chromatography purity. Eicosanoids used for primary standards in standard curves as well as their deuterated analogs were from Cayman Chemicals and Biomol (Enzo Life Science, Farmingdale, NY, USA). For extraction, 5 mg of brain tissue (entorhinal cortex) was supplemented with a cocktail consisting of 26 deuterated internal standards, homogenized with 500 μL 10% methanol on ice and briefly sonicated. Samples were then purified by solid phase extraction on Strata-X columns (Phenomenex, Torrance, CA, USA) following the activation procedure provided by the distributor. Samples were eluted with 1 mL of 100% methanol, the eluent was dried under vacuum and dissolved in 50 μL of buffer A consisting of 60/40/0.02 water/acetonitrile/acetic acid = 60/40/0.02 (v/v/v) and immediately used for analysis.

#### Reverse-phase liquid chromatography and mass spectrometry

Eicosanoids were analyzed as previously described (Quehenberger *et al*., 2011). Briefly, eicosanoids were separated by reverse-phase chromatography using a 1.7 μm 2.1 × 100 mm BEH Shield Column (Waters, Milford, MA, USA) and an Acquity UPLC system (Waters). The column was equilibrated with buffer A, and 5 μL of sample was injected via the autosampler. Samples were eluted with a step gradient to 100% buffer B consisting of acetonitrile/isopropanol = 50/50 (v/v). The liquid chromatography effluent was interfaced with a mass spectrometer, and mass spectral analysis was performed on an AB SCIEX 6500 QTrap mass spectrometer equipped with an IonDrive Turbo V source (AB SCIEX, Framingham, MA, USA). Eicosanoids were measured using multiple reaction monitoring (MRM) pairs with the instrument operating in the negative ion mode. Collisional activation of the eicosanoid precursor ions was achieved with nitrogen as the collision gas, and the eicosanoids were identified by matching their MRM signal and chromatographic retention time with those of pure identical standards.

#### Quantitation of eicosanoids

Eicosanoids were quantified by the stable isotope dilution method. Briefly, identical amounts of deuterated internal standards were added to each sample and to all the primary standards used to generate standard curves. To calculate the amount of eicosanoids in a sample, ratios of peak areas between endogenous eicosanoids and matching deuterated internal eicosanoids were calculated. Ratios were converted to absolute amounts by linear regression analysis of standard curves generated under identical conditions. Currently, we can quantify over 150 eicosanoids at sub-fmole levels.

### RNA analysis

The nCounter GX Mouse Inflammation Kit (Nanostring, Seattle, USA) was used to measure a comprehensive set of 179 inflammation related mouse genes and six internal reference genes. RNA was isolated from occipital, parietal, and temporal cortices using the RNeasy mini kit (Qiagen, Gaithersburg, MD, USA) following the manufacturer’s instructions and RNA analysis performed by Nanostring.

### Toxicological analysis

Tissues from wild-type control and fisetin-treated mice were sent to Colorado Histo-Prep (Fort Collins, CO, USA) for histopathological evaluation. A 2-day acute toxicity assay was performed by Absorption Systems (San Diego, CA, USA). The Ames test using strain TA100 was performed as described (Maron & Ames, 1983).
